# Unemployment Status Subsequent to Cancer Diagnosis and Therapies: A Systematic Review and Meta-Analysis

**DOI:** 10.3390/cancers15051513

**Published:** 2023-02-28

**Authors:** Martina Chimienti, Giustino Morlino, Fabio Ingravalle, Antonio Vinci, Emilio Colarusso, Carolina De Santo, Valeria Formosa, Lavinia Gentile, Grazia Lorusso, Claudia Mosconi, Martina Scaramella, Virginia Rosca, Elena Veneziano, Francesco Torino, Leonardo Emberti Gialloreti, Leonardo Palombi

**Affiliations:** 1 Post-Graduate School of Hygiene and Preventive Medicine, University of Rome “Tor Vergata”, 00133 Roma, Italy; 2 Hospital Health Management Area, Local Health Authority “Roma 6”, 00041 Albano Laziale, Italy; 3 Hospital Health Management Area, Local Health Authority “Roma 1”, 00133 Roma, Italy; 4 Department of Systems Medicine, Medical Oncology, University of Rome “Tor Vergata”, 00133 Roma, Italy; 5 Department of Biomedicine and Prevention, University of Rome “Tor Vergata”, 00133 Roma, Italy

**Keywords:** unemployment, cancer, cancer therapies, disability

## Abstract

**Simple Summary:**

In the future, numbers of cancer survivors will increase due to early detection and new therapies, but it is important to consider the long-term consequences of disease and treatment. The purpose of this study is to assess the impact of cancer and treatment on the employment status of cancer survivors. Implementing a strict selection of articles to be analyzed, in order to mitigate the interference of publication and selection bias on the final results, we highlighted how cancer, treatments, and related disabilities are risk factors for unemployment. We believe that the negative influence of cancer on people’s lives, including in terms of employment status with consequential fallout for morbidity and mortality, needs to be addressed by promoting health and social welfare support programs from diagnosis to follow-up, substantiating patient involvement in treatment choices.

**Abstract:**

The purpose of our study is to examine whether cancer and treatments are associated with job loss or changes in employment status. Eight prospective studies were included in the systematic review and meta-analysis, with a population aged 18–65 years, analyzing treatment regimen and psychophysical and social status in post-cancer follow-up of at least 2 years. In the meta-analysis, a comparison was made between recovered unemployed cases and cases from a standard reference population. Results are summarized graphically using a forest plot. We showed that cancer and subsequent treatment are risk factors for unemployment with an overall relative risk of 7.24 (lnRR: 1.98, 95% CI: 1.32–2.63) or for change in employment status. Individuals undergoing chemotherapy and/or radiation treatment and those with brain and colorectal cancers are more likely to develop disabilities that negatively affect the risk of unemployment. Finally, variables such as low level education, female sex, older age, and being overweight before starting therapy are associated with higher risk of unemployment. In the future, it will be necessary for people with cancer to have access to specific health, social welfare, and employment support programs. In addition, it is desirable that they become more involved in their choice of therapeutic treatment.

## 1. Introduction

The International Agency for Research on Cancer (IARC) *Biennial Report 2020–2021* [1] estimates a 60 percent increase in cancer cases over the next 20 years. This increase, accompanied by substantial therapeutic improvements, will increase the number of long-term survivors. 

Although, the majority of long-term cancer survivors are beyond retirement age [2], consideration must be given to the rising retirement age in some industrialized countries [3,4], and also the onset of demographic and epidemiological transitions in developing countries, comparable to those observed in the baby-boomer generation in developed countries [5,6], which will result in a large number of cancer survivors of working age.

These conditions result in the growing social importance of the possible loss of work in people who receive a diagnosis of cancer during working age. From this perspective, unemployment should be considered not only a socio-economic issue, but also a public health problem. A systematic review and meta-analysis [7] showed that the lifetime risk of death from all causes was 63% higher among unemployed people.

Some recent research suggests that people with cancer are more likely than others to lose their jobs. For example, a systematic review published in 2010 [8] found that only 63.5% of cancer survivors went back to their jobs. This condition was associated with certain characteristics such as employer accommodation and flexible working. Another study showed that cancer survivors were more frequently unemployed than healthy controls [9]. However, large differences were found among different cancers, while in some cases, such as blood or prostate cancers, these differences were absent. Furthermore, in this case the type of treatment was not considered.

Other studies have been carried out, looking for possible associations between unemployment and a single type of cancer treated with one or more therapies. For example, a systematic review and meta-analysis published in 2018 focused on elements that could be correlated with unemployment after surgical treatment of breast cancer, such as high psychological job demands or lower educational attainment [10]. Other authors [11] performed a systematic review in which employment rates of Hodgkin lymphoma survivors were lower than prior to diagnosis, although they were similar to those of the general population.

Several factors may be associated with keeping a job after a cancer diagnosis, such as professional status and income, socio-demographic characteristics, the type of cancer and related treatment. All these factors might interact in decreasing employment rates by 25–35% [12].

Evidence of job loss has been also observed in adults who contracted cancer during childhood. For example, a systematic review [13] showed a higher risk of unemployment in survivors of central nervous system tumors diagnosed during childhood.

A systematic review and meta-analysis published in 2020 evaluated “long-term work retention after treatment for cancer” [14], and described the association between cancer survivorship, employment, and various related risk factors. In their systematic review, the authors revealed a possible relationship between chemotherapy and work retention.

Despite these advancements, cancer-related unemployment remains a controversial topic, especially in relation to type of cancer, treatment, and socio-economic conditions of the person at the time of diagnosis [8,11,15,16]. For this reason, it is important to clarify the possible effects of cancer and subsequent cancer treatments on job loss. Understanding this may allow planning and development of public health interventions aimed at preventing unemployment in this category of people, to improve their quality of life [17] and reduce healthcare costs and public spending. 

Our research aims to quantify the risk of unemployment for cancer survivors and populations under treatment by identifying relative risk by comparing data from selected articles with reference populations, through the use of official databases. Furthermore, only prospective studies were selected, as articles of this type help to reduce the impact of information bias on the final result. These choices represent a methodological novelty in the scientific literature compared with previous research.

### Objectives

This study aimed to examine whether cancer and its consequences, such as the use of antineoplastic therapies, can be associated with job loss or changes in employment status after a follow-up period of at least two years, which is the theoretical period after diagnosis when there remains a high chance of active disease and necessity for treatment. Therefore, the null hypothesis (H0) is that cancer has no effect on occupational status.

## 2. Materials and Methods

The systematic review and meta-analysis were performed following an a priori designed protocol, and reported in accordance with the 2020 Preferred Reporting Items for Systematic Reviews and Meta-Analyses (PRISMA) guidelines [18].

The research question was framed using PICO (Patients, Intervention, Comparison, and Outcome). PICO items were defined as per Table A1.

### 2.1. Study Protocol and Registration

The protocol of our article is registered on Prospero with ID: CRD42022383544.

### 2.2. Information Sources and Search Strategies

The MEDLINE and Scopus databases were searched electronically, in April 2022, using combinations of the relevant medical subject headings, key words, and word variants for cancer, therapy, and employment status. Table A2 shows the search strings and the filters used for search optimization.

### 2.3. Selection Process

Two different authors independently screened paper titles and abstracts in each database: AV and GM for Scopus, FI and MC for PubMed/Medline.

Disagreements were discussed between the authors and resolved by consensus or by recourse to a third author (EC). Studies were then labeled for inclusion or exclusion.

**Figure 1:**
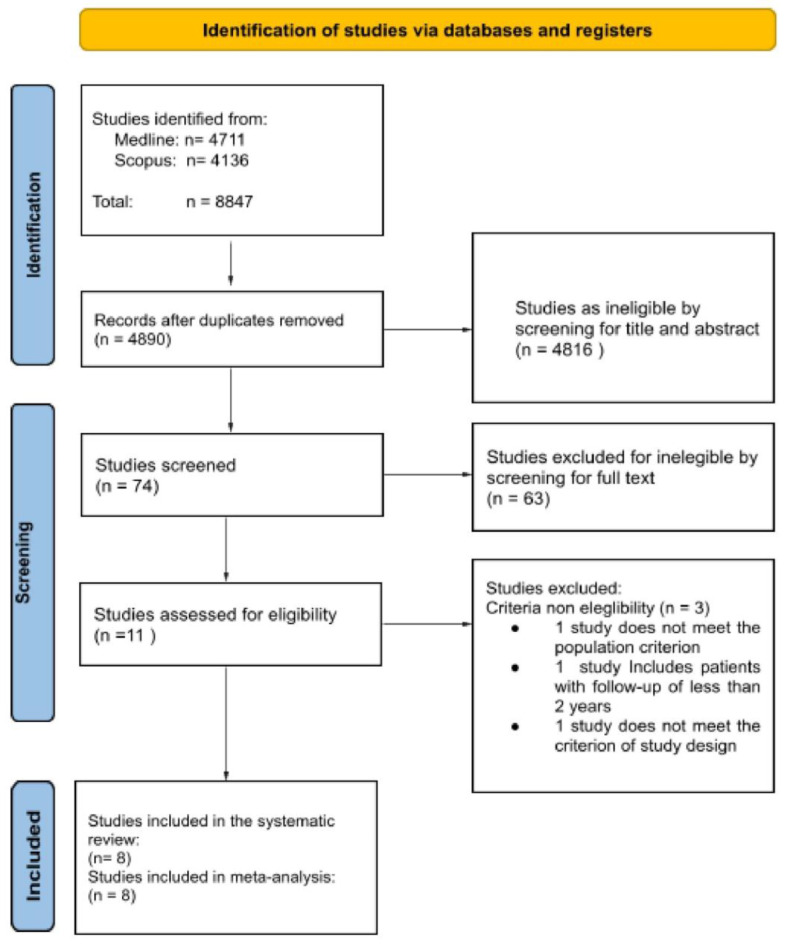
Workflow process.

Each article meeting the eligibility criteria was considered for subsequent qualitative synthesis; duplicate records and articles that included an exclusion criterion were removed. Particularly, we removed studies which included only a specific subset of workers, which focused on unhealthy populations or only on specific population subsets (e.g., blue-collar or retired workers), or which measured only short-term outcomes. Then, two different authors (CDS, MC) independently screened the entire content of the articles and studies in an analogous manner to the selection process for title and abstract. Similarly, disagreements were discussed between the authors and resolved by consensus or by resorting to a third author (VF). This selection process is summarized in a flow chart (Figure 1).

#### 2.3.1. Eligibility Criteria

Studies were included or excluded from the review according to the criteria below.

Inclusion criteria:

- Study design

     Prospective studies;
     Studies presenting one or more measures of association.

- Exposure

     Any regimen options;
     At least two-year cohort follow-up.

- Outcome

     Change in employment status.

- Publication type

     Primary studies published in peer-reviewed journals;
     Non-peer reviewed publications (e.g., government reports), if publicly available;
     In English language or available in English translation;
     Published after the year 2000.

Esclusion criteria:

- Exposure

     Unemployable individuals, e.g., due to disease, disability, or age;
     Population limited to a specific occupation.

- Study design

     Cross-sectional (prevalence) studies without prospective elements;
     Case reports and case series;
     Retrospective studies;
     Prospective studies without measures of association and/or without confidence intervals (or data enabling their calculation);
     Studies measuring short-term effects (less than two years of follow-up) of therapies on employment status (e.g., time-series analyses).

- Publication type

Abstracts with no full text available; 

Studies whose results have been superseded and replaced by later reports of the same study (including conditions in which the reports presented a mixture of updated and non-updated results).

Out of all the selected studies, only those whose reported data allowed the required calculations to be performed were also included in the meta-analysis.

#### 2.3.2. Data Collection Process

For data extraction, we used two different pairs of authors (CDS and MS, EV and GL), who collected data independently. Studies with missing or unclear data were excluded from the meta-analysis but included in the synthesis. Disagreements were discussed between the authors and resolved by consensus or by resorting to a fifth author (GM).

For every study included in the meta-analysis, following data were retrieved:

- Number of participants;
- Observation interval (in years);
- Treatment type;
- Country;
- Type of cancer;
- Number employed;
- Number unemployed.

#### 2.3.3. Study Risk of Bias Assessment

In order to assess the risk of bias and confounding, the RTI item bank (RTI) of the Agency for Healthcare Research and Quality was employed. This tool has been specifically designed for evaluating observational studies of interventions or exposures [19]. It consists of several questions with dichotomous Yes\No answers, which guide the reviewer’s evaluation of the study. The evaluation was performed by two different authors in a double-blind manner, as for the previous selections (EV, VR). Any disagreements were resolved by consensus or entrusted to a third author (LG).

#### 2.3.4. Effect Measures

The effect size used for quantitative syntheses was the pooled risk ratio (RR). Information not used in the meta-analysis was considered for narrative synthesis.

#### 2.3.5. Synthesis Methods

We selected for narrative synthesis all studies that met the inclusion criteria and were therefore included in the meta-analysis.

To quantify the relative risk (RR) of experiencing unemployment after a cancer diagnosis, we compared the retrieved unemployed cases with incident cases from a standard reference population, calculated as follows:

Mean unemployment incidence rate in countries and period of time pertaining to the included studies were estimated through data retrieved from Eurostat (for EU countries) [20] or the World Bank (for non-EU countries) [21]. Using this data, after adjustment for gender, the expected numbers of employed and unemployed people could be estimated for the period. Such numbers represented theoretical control populations with the same size and composition of those included in the studies. Adjustment for age was not possible; however, all study data referred to the workforce-aged population only. Meta-analysis was then performed by comparing observed and expected unemployment incident cases for each conducted study.

Results are reported narratively, graphically, and summarized by means of tables where appropriate.

Given the obvious heterogeneity in the designs of the retrieved studies, as well as the fact that each study observed different populations, we chose to perform the meta-analysis using a pooled REML (random-effects restricted maximum likelihood) model of the risk ratios, without performing any formal heterogeneity-estimation test. This model was chosen because it was considered the most suitable due to the heterogeneity of the data, with the aim of reducing its weight in the final result. However, to estimate the proportion of variance due to true effects rather than sampling error, statistics were nonetheless calculated. Additionally, to investigate possible causes of heterogeneity in the study results, reported secondary outcomes and objectives were also considered. Finally, leave-one-out analysis was performed to investigate the possible presence of overstated effect sizes, which can distort the overall results.

Statistical analysis and graph drawing were performed using Microsoft Excel™ Professional Plus 2016 (Italian anguage) and Stata^®^ SE v.17.0 software. Results were synthesized and are tabulated in a summary of findings.

#### 2.3.6. Reporting Bias Assessment

To assess the potential role of publication bias related to outcomes with data available from ten or more studies, the funnel plot method was applied. Because this meta-analysis was designed to explore binary primary outcomes, funnel plot asymmetry was assessed by means of Egger’s linear regression test [22].

#### 2.3.7. Certainty Assessment

The GRADE approach was employed to assess the certainty of evidence [23,24]. Risk of bias was assessed using the Agency for Healthcare Research and Quality (RTI) tool [19]. Inconsistency was assessed by calculating the statistic, as described by Higgins and Thompson [25]. Indirectness was excluded from the protocol, selecting only studies with well-defined outcomes. Precision was assessed by pooling the estimates using two different models: an FE model weighting the data by within-study variance, and an RE model weighting the data by between-study variance. The *p* values and confidence intervals (CIs) were calculated for each model in order to highlight any accuracy issues.

## 3. Results

### 3.1. Study Selection

The search and selection process is illustrated in the flowchart (Figure 1). A total of 8847 studies were retrieved from the databases. Following the screening sessions, 74 studies were selected for full-text evaluation, of which 66 studies were excluded.

### 3.2. Study Characteristics

According to the GRADE recommendations, characteristics of the included studies and summaries of their findings are presented in Table 1.

### 3.3. Risk of Bias in Studies

**Figure 2:**
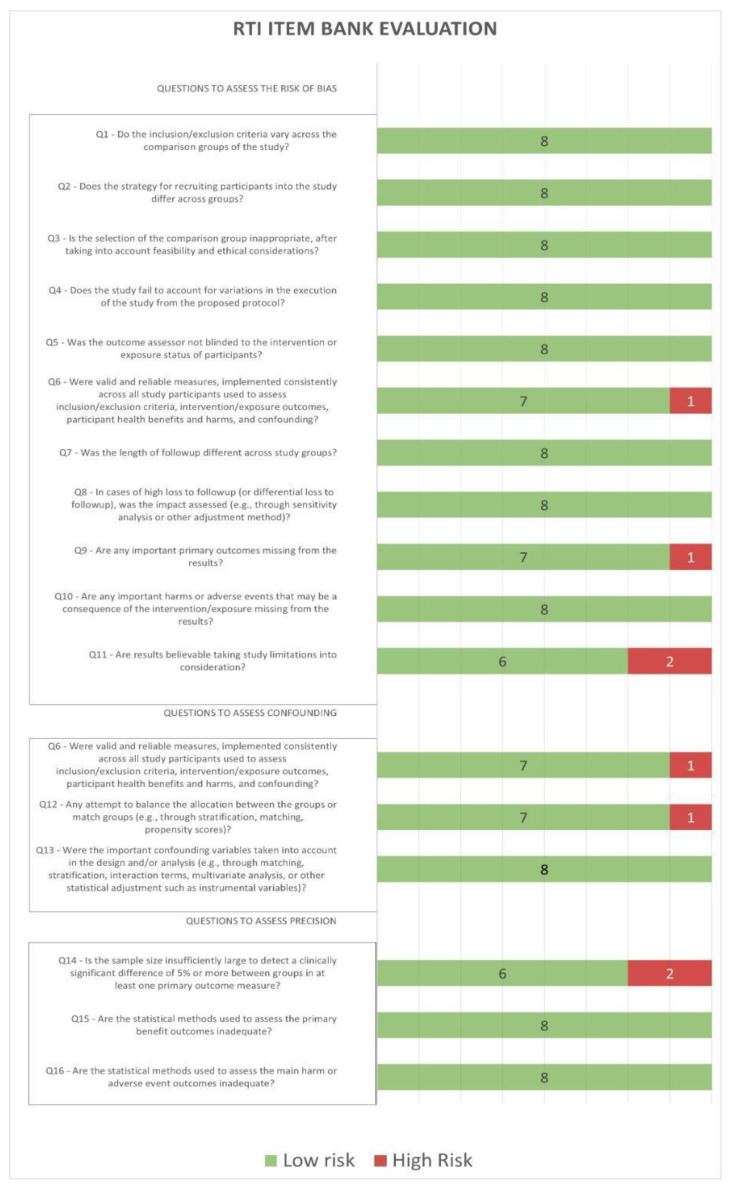
RTI quality and risk of bias in studies included in the meta-analysis.

RTI was used for the evaluation of the risk of bias and confounding. The method consists of 16 Yes/No questions, designed to help reviewers in evaluating a study’s potential risk of bias. Analysis of the eight included studies is reported in Table 2 and graphically summarized in Figure 2.

### 3.4. Results of Individual Studies

#### 3.4.1. Primary Result

A previous research group [12] conducted a study in France, interviewing patients diagnosed with cancer in 2010 after a 5-year follow-up. After 5 years, 82% of the interviewees were still employed; however, only 55% kept the previous working hours, and 26% reduced their hours compared with their job situation at diagnosis. Among the 18.4% no longer employed, 0.9% were retired, 5.9% unemployed, 2.0% were homemakers, and 8.7% on invalidity benefit. In addition, out of all cancer survivors, 30% were affected by chronic neuropathic pain; consequently, some of them reduced their working hours, while others lost their jobs. 

Another study analyzed breast cancer survivors [32]. It examined cancer survivors’ return to work after treatment using one of the largest cohorts of breast cancer survivors available at that time, the CANcer TOxicity cohort. Two years after breast cancer diagnosis, 398 patients (21.3%) had not yet returned to work. 

In 2021, a study conducted in Taiwan used data collected [26] from the National Health Insurance Research Database (NHIRD), the Labor Insurance Database (LID), and the Taiwan Cancer Registry (TCR). Data included information about 2451 workers diagnosed with liver cancer between 2004 and 2010. The primary outcome was the return-to-work rate 1–5 years after liver cancer diagnosis. In the 2nd year, 1504 workers had returned to work, 550 had died, and 397 were unemployed; in the 5th year, 1123 workers had returned to work, 940 had died, and 388 were unemployed. 

A prospective cohort study was conducted in Germany [28], analyzing the neuro-oncological and functional outcomes of 58 patients with newly diagnosed grade II and III gliomas (WHO classification). The patients underwent surgery between August 2012 and June 2018. The impact of the disease on work and socioeconomic status was retrospectively assessed through questionnaires, andthe median follow-up was 43.8 months (>3 years). After treatment, 41 patients (70.7%) resumed a working life, while 17 patients (29.3%) did not.

Another prospective cohort study was performed [30] in the Netherlands. In this case, 76 patients who underwent treatment for rectal cancer were analyzed through questionnaires, which assessed functional outcomes (physical symptoms, functional autonomy, sexual activity) and their current employment status after therapy (surgical, radiotherapy, or multimodal treatment). Overall, 14 patients lost their jobs during the follow-up period, 11 retained theirs, and 10 resumed employment in lighter work.

In the Netherlands, researcher [31] performed a prospective cohort study from 2014 to 2017 analyzing outcomes after breast cancer. A population of 939 patients was analyzed through four questionnaires before and after undergoing treatment for breast cancer (only patients who responded to at least two questionnaires were considered for study purposes). Analyzed therapies included systemic neoadjuvant therapy (chemotherapy or chemotherapy + immunotherapy), radiation therapy, surgical treatment, adjuvant chemotherapy treatment, and adjuvant endocrine treatment. The questionnaire was the Work Ability Index (WAI), which analyzes type of work, work activity, hours of work, intensity of work activity, days away from work, and mental and physical work capability in relation to the disease, including possible consequent work limitations. At the end of the follow-up period, out of 319 responders, there were 116 unemployed individuals, while 203 people had retained their jobs.

A cross-sectional study with prospective elements [29] carried out in Norway from 2012 to 2013 analyzed 18-year-old lymphoma survivors who were still alive in 2011, and who had undergone high-dose chemotherapy with autologous stem cell transplantation. At follow-up, 47% of the interviewees were employed and male, while the unemployed group consisted mainly of older adults and women. Employed people tended to presented with a better quality of life, and, furthermore, the unemployed experienced greater problems related to depression and comorbidities.

In 2022, researchers [27] longitudinally analyzed 4852 ≥ 18-year-old patients who underwent craniotomy for excision of malignant brain tumors diagnosed between 2011 and 2017. Among them, 12.3% (595) lost their jobs within one year after surgery.

#### 3.4.2. Secondary Results

Several included studies also analyzed various secondary outcomes. These results can be ascribed to two macro areas of health determinants: unmodifiable (e.g., age, gender) and modifiable factors (e.g., education, bodyweight, therapy received).

Therapy: Table 3 describes the main disabilities and adverse effects related to cancer treatments. A cohort study conducted in Taiwan in 2021 [26] showed that 98% of those who returned to work and 94.9% of those who did not had received surgical treatment (*p* < 0.05). The Taiwanese study demonstrated that surgical treatment had a positive effect and increased the rate of return to work within 5 years. In contrast, a 2022 South Korean cohort study [27] of patients with malignant brain tumors showed that surgical treatment had a negative impact on employment status, and chemotherapy was associated with a lower rate of 2-year return to work. Based on the Barcelona Clinic Liver Cancer (BCLC) staging system, surgical treatment is usually performed in patients with early-stage disease, while chemotherapy is usually used in advanced cancer. The side effects of chemotherapy that make it difficult to return to work should also be considered. In another study, patients were found to be more likely to return to work after extensive resections (removal of 90% or more of the preoperative tumor volume) compared to partial resections (76% vs. 44%; *p* = 0.106) [28]. A French study [32] analyzed the risk of non-return to work: the odds ratio of patients undergoing mastectomy vs. partial surgery was 1.46 (95% CI: 1.07, 1.99), while the OR of those undergoing adjuvant anti-HER2 therapy vs. no therapy was 1.71 (95% CI: 1.21, 2.42). Another study reported that therapy might influence ability to work. Moderate or poor work ability was reported by 24% of patients 30 months after diagnosis. In particular, axillary lymph-node dissection, (neo)adjuvant chemotherapy, and locoregional radiotherapy were associated with reduced work ability. After 30 months, 18% of employed individuals reported having reduced their working hours, having made substantial changes to their work patterns, or being unable to work. Only 17% of survivors did not report encountering any limitations at work [31].

Education: Two French studies analyzed the educational levels of individuals with cancer. In both cases, an association emerged between low educational qualifications and unemployment. One study reported that the employment rate was higher in people with higher educational qualifications working in the tertiary sector, or who had managerial jobs in public or private sectors. Overall, 88.6% of those with at least a high school diploma maintained post-cancer employment, compared with 73.2% of people with a lower middle school diploma and 54.8% of people without any degree [12]. The second study calculated that the ORs of non-return-to-work were significantly higher in patients with a lower education level (primary school vs. college or postgraduate) (OR = 2.96; 95% CI: 1.56, 5.60) [32]. 

Bodyweight: A single French study [32] analyzed whether bodyweight influenced return to work in cancer survivors. Presented data indicate that excess bodyweight at the time of breast cancer diagnosis can be a significant obstacle to returning to work, and that overweight and obese patients represent a group at higher risk of unemployment. The rate of non-return to work was 17.7% among underweight or normal-weight patients, compared to 27.4% among overweight or obese patients (*p* < 0.0001). The frequencies of women who did not return to work were 121/434 (27.9%) for women with a BMI of 25.0–29.9 kg/m^2^, 49/169 (29.0%) for those with a BMI of 30.0–34.9 kg/m^2^, and 19/86 (22.1%) in patients with BMI ≥ 35.0 kg/m^2^. 

Quality of life: A Korean nationwide population-based cohort study showed that nearly half of patients (46.9%) who underwent a craniotomy for excision of brain tumors experienced a deterioration in their quality of life (QOL) in the first year after surgery: 1329 (27.4%) earned less and 844 (17.4%) developed new disabilities. Being male (OR: 1.17, 95% CI: 1.04–1.31; *p* = 0.009), reoperation within 1 year (OR: 1.36, 95% CI: 1.02–1.79; *p* = 0.033), and longer stay (OR: 1.02, 95% CI: 1.02–1.03; *p* <0.001) were significantly associated with increased QOL deterioration [27]. Another study reported that the unemployed had more problems related to depression (OR: 2.62, 95% CI: 1.32–5.21; *p* < 0.01) and type D personality (OR: 2.42, 95% CI: 1.37–4.29; *p* < 0.05) than the employed, confirmed by univariate and multivariate analyses [29].

Gender: Several studies reported how gender might affect employment after a cancer diagnosis. The results are inconsistent. According to a Netherlands study on rectal cancer [30], 31% of affected women lost their jobs, compared to 11% of men (*p* < 0.01) Another study reported that the employed group included significantly more males (68% vs. 32% *p* value < 0.001), while among the unemployed the ratio was reversed with most females (55% vs. 45% *p* value < 0.001) [29]. In a 2021 Taiwainese study, the male gender was positively associated with return to work (*p* < 0.05) [26]. On the other hand, a German study [28] found that gender was not significantly statistically associated with patients’ ability to return to work. Similarly, a French survey conducted in 2018 [12] showed no significant difference between men and women in terms of the rate of employment preservation (78.8% vs. 82.2%; *p* = 0.299).

Age: A study performed in Taiwan showed that the mean age of the return-to-work group was 50.5 ± 8.8 years, and the mean age of the non-return-to-work group was 52.7 ± 9.5 years (*p* < 0.05). In the same study, univariate and multivariate correlations suggested that a young age was positively related to return to work (*p* < 0.05) [26]. The analysis performed on a German group in 2020 [28] showed that patients’ age was significantly associated with the ability to return to work (94.1% among patients <40 years vs. 37.5% among patients ≥40 years old). Undergoing surgery while being younger than 40 was associated with a higher probability of return to work (*p* < 0.01). Another study reported that the level of employment preservation was significantly higher among survivors aged 18–39 at cancer diagnosis and among those aged 40–49 than among those aged 50–54 (81.5%, 85.4%, and 72.9%, respectively; *p* < 0.001) [12]. According to a 2016 Norwegian study [29], employed subjects were significantly younger than the unemployed (mean age at follow-up: 50.4 (11.1 SD) and 55.6 (12.5 SD) years, respectively (*p*-value < 0.001). 

### 3.5. Meta-Analysis

All eight included studies were selected for the meta-analysis. Their characteristics are reported in Table 3.

An overall total of 11,495 people was included in the analysis, with studies conducted in six different countries/regions: Taiwan (1), South Korea (1), Germany (1), The Netherlands (2), France (2), and Norway (1).

All studies were continued for several years, with a follow-up period ranging from 2 [27,29,32] to 5 years [12,26,30]. As shown in Figure 2, all these studies were of high quality. Seven studies were observational cohort studies, while one was cross-sectional (but was treated as an observational study for the work-status analysis, with two disclosures, the first at the time of diagnosis and the second at the time of interview) [29].

Cancer types observed in the studies included liver cancer, brain tumors, lymphomas, colorectal cancer, and breast cancer. In seven studies, the surgical approach was associated with chemotherapy and/or radiotherapy; in one case (lymphoma) the treatment was chemotherapy and autologous stem cell transplantation [29].

Data sources were national databases [26,27], clinical data [28], telephone interviews [12], or questionnaires administered to patients [29,30,31,32].

**Figure 3:**
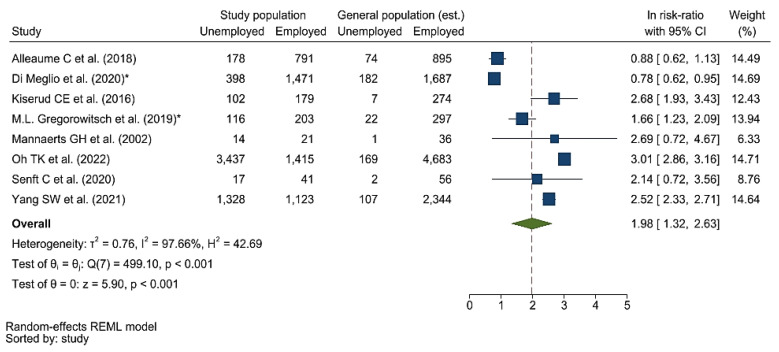
Forest plot of included studies. *: population composed of women only [12,26,27,28,29,30,31,32].

Meta-analysis results from the eight included studies were summarized in a forest plot (Figure 3). RE model results show that undergoing cancer treatment is a risk factor for a change in employment status or unemployment, with an overall relative risk of 7.24 (ln RR = 1.98; 95% CI: 1.32, 2.63).

#### 3.5.1. Reporting Biases

Possible publication bias could not be adequately assessed, neither using the funnel plot nor Egger’s regression test, due to the small number of selected studies.

#### 3.5.2. Certainty of Evidence

The authors believe that, for this type of study, instead of focusing on finding a “true” value (as is the case in FE models and unweighted analyses), the use of a random-effects model is most appropriate, because the outcome is conceptually the most likely value from a distribution of results. This choice should be considered optimal because the individual studies included were from heterogeneous populations and did not follow exactly the same protocol, although they were sufficiently similar to be included in the statistical analysis.

Heterogeneity among the studies was calculated using the I^2^ test. Very high heterogeneity (>97%) was found across the included studies.

**Figure 4:**
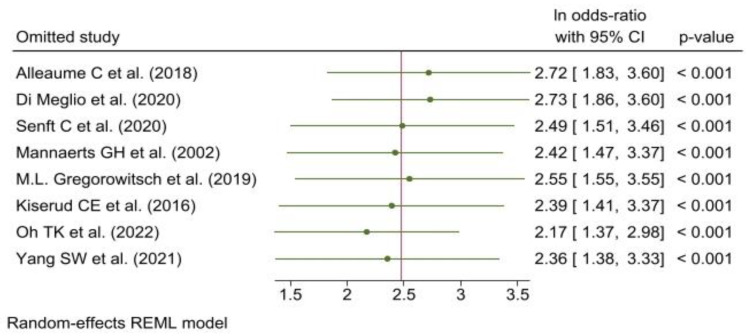
Leave-one-out analysis [12,26,27,28,29,30,31,32].

Leave-one-out analysis did not show any study influential in determining the overall risk ratio or 95%CI. (Figure 4).

## 4. Discussion

Our meta-analysis showed that cancer and subsequent therapeutic treatment places an individual at a risk of unemployment within 5 years after diagnosis, with an overall relative risk of 7.24 (ln RR: 1.98, 95% CI: 1.32–2.63). 

This finding indicates that the livelihood of experiencing a change in employment status after becoming ill with cancer are significantly higher than in the general population, regardless of the cancer’s type or site, the degree of malignancy of the disease, the evolution of the clinical picture, and the treatment administered.

Meta-analyses conducted in 2020 [19] and 2011 [8] confirm the above, reporting that, regardless of geographic area, site of origin, and type of cancer, patients who have contracted cancer and undergone treatment have a higher risk of becoming unemployed than the general population (72% chance of keeping a job between 2 and 2.9 years in the first study [19], between 26% and 53% of cancer survivors lost their jobs within 72 months of diagnosis in the second [33]). In addition, the decision to adopt a strict selection of studies, excluding retrospective studies, ensured limited interference by possible information bias in the study results. 

### 4.1. Results in Context

Patients with tumors at specific sites, such as brain tumors [27,28] or rectal/colon tumors [30], have a higher propensity for permanent disability and thus a lower propensity for re-employment than other treated neoplastic forms; this finding reinforces the observation that the main culprit for occupational change is disability acquired during the course of the disease or as a result of therapy. As confirmed by numerous studies [34,35,36], most patients with brain tumors experience neurocognitive impairments during the course of the disease or secondary to therapeutic treatment. These impairments can persist even years or decades after diagnosis and undermine patients’ work and daily living skills. With regard to colon/rectal cancer, there is a clear association with unemployment, and 40% of the sample analyzed left the workplace permanently within thirty months after diagnosis [33].

In agreement with the literature, it is worth highlighting how some therapies correlate with higher rates of unemployment and work disability. As evidenced by six out of eight of our selected studies [12,26,28,29,30,32], patients who have undergone chemotherapy treatments exhibit adverse health effects such as mental and physical fatigue, cognitive impairments of memory and attention, and mood alterations such as anxiety, social inhibition, and depression, resulting in reduced work performance and higher risk of unemployment [10,37,38,39].

Radiation therapy also appears to be related to the onset of disabilities that may lead to changes in employment status: the onset of chronic neuropathic pain (CNP) appears to be more related to certain therapies such as chemotherapy (36.3%) and radiation therapy (32.8%), with locoregional radiation therapy in particular appearing to be more implicated than local radiation therapy [40]. In addition, radiotherapy negatively affects patients’ cognitive function, in both the short and long term [41]. 

Regarding surgical treatments, contrasting positions are revealed: in breast cancers, conservative treatments have shown higher rates of return to work, probably related to less invasiveness and reduced chance of complications for patients [10,32]; in contrast, for some brain cancers, radical surgery treatments are associated with higher rates of return to work [28,42].

Despite the fact that some studies analyzed in the review [12,32] show that people with cancer who have lower levels of education have higher rates of unemployment, thus underscoring how social components may impact employment status, in line with the literature [14] we must consider that these individuals have more physically demanding occupations on average and consequently experience greater work limitations related to physical fatigue or disability post-disease.

One of the studies revewed [32] hypothesized that high BMI may be a risk factor for unemployability in cancer survivors, which might be explained from both a psychophysical and social perspective. Indeed, the literature [43] argues that overweight and obese individuals may be more susceptible to adverse effects of antineoplastic treatments and on average develop worse physical and psychological disabilities.

In addition, more than one study [44,45] has stated that obese people have greater difficulty in obtaining employment and regaining it after losing it, possibly due to possible discrimination from employers encountered because of their appearance, experiencing longer periods of unemployment on average. Aa additional factor capable of having an effect on employment status is the sex of the patient.As shown by some studies [26,29,30], female patients are more likely to experience reduction in work intensity, job loss, or unemployment. In contrast, other researchers [12,28] reported no statistically significant correlation between sex and employment status in the samples they analyzed.

In view of the contradictory findings, our studies in agreement with the literature suggest that the causes of gender differences in job re-entry are social in nature. In fact, multiple studies [46,47,48] have pointed out that certain factors predominantly burden female subjects: a hostile work environment, in terms of support from colleagues and employers, inflexible working hours, irreconcilable with the demands of care and convalescence, and discriminatory perceptions of one’s illness. 

However, we cannot completely rule out the consequences of adverse effects produced by cancer and antineoplastic therapies on biologically different genders. The final factor for analysis is age, especially considering the multiple influences it has on people’s occupational status.

Younger patients with cancer are more likely to return to work, compared with older individuals [12,26,28,29]. Despite the fact that this finding is cited several times in the literature [14,49], it is not possible to determine whether this observation is a consequence of increased sensitivity of older patients to the adverse effects of treatment, or relates to the influence of the labor markets of the countries considered in the articles.

### 4.2. Limitations of Included Studies

Precisely because of the rigidity of the criteria used, this study has some limitations: first of all, the small number of research studies selected probably relates to the higher cost of prospective studies compared with other types of research, limiting their implementation and thus their retrieval.

Limitations associated with the prospective nature of the included studies include the marginality with which some topics were treated: in some studies, unemployment was not the primary outcome, and in others, unemployed cohorts were not adequately compared with regard to certain determinants of health that have a bearing on employment status, such as gender, educational attainment, age, social status, etc.

An additional obstacle was the high heterogeneity of the selected studies, differing in their reporting of cancer types, sites, staging, and therapies. Despite the fact that antineoplastic treatments are universally standardized for the above criteria, with a comparable psychophysical and social effecton the occupational status of patients, it is not possible to show a precise correlation reflecting how individual therapy affects occupational status. The individual studies analyzed failed to quantitatively demonstrate how individual therapy can impact employment status. It will be important to conduct further studies in this regard.

The cultural impact of each country’s attitudes to illness and unemployment should also be considered in relation to the huge differences between Western and Asian countries. 

### 4.3. Limitations of the Review Methods

The studies included in the systematic review and meta-analysis showed a high degree of heterogeneity, due to the different types of cancer studied and the different study designs, which were generally observational in nature and of long duration. 

A standardized healthy population, estimated from rates available from authoritative and certified sources, was assumed in order to perform the relative risk calculation for comparison with the study population. This choice was made to ensure repeatable comparison with the study populations included in meta-analyses.

In addition, the population was stratified by sex, in accordance with selections in specific studies [31,32], however, it was not possible to stratify by age group in relation to the types of cancer studied, because the World Bank database [21] does not provide unemployment stratification by age.

Another limitation is the inability to stratify the extracted data for possible confounding factors, caused by the fact that the studies reviewed did not analyze or only partially analyzed possible confounding factors. In addition, the studies available in the scientific literature identified neither the type of employment relationship, other characteristics of the employment relationship, nor any benefits of maintaining employment.

However, we believe that these limitations, while present, do not invalidate the results of the current study, as the estimate obtained is concordant with other similar types of employment, and no single study taken individually was decisive for the final meta-analysis result.

## 5. Conclusions

This study revealed a significant correlation between cancer, cancer-related therapies, and job loss (RR: 7.24), therefore, despite the incontrovertible efficacy and usefulness of antineoplastic therapies in the care and treatment of the cancer patients, especially in the early stages of the disease, it is important to take note of the possibility of long-term functional impairment and subsequent psychophysical disabilities following treatment, which could translate to work-related consequences for the patient. Socioeconomic variables and work culture in each state can have strong influences on such effects.

According to IMF (International Monetary Fund) standards, all the countries considered in this paper have high GDP per capita PPP [50], and on their average citizens have good economic and working conditions. As further evidence of this, all of the selected states in the LEGATUM prosperity index (a scale that takes into account several factors including health, economic growth, education, wellbeing, and quality of life) occupy high positions in both the overall rankings and with regard to economic wellbeing [51].

On the basis of the analysis, with the understanding that the socioeconomic component cannot be excluded, it can be hypothesized that the main agent responsible for the change in the employment status of the studied population was the harm produced by the cancer itself, the therapies, and related adverse effects.

Corroborating this hypothesis, the study conducted in South Korea [27] reported that patients who undergo new operations and those with longer average hospital stays experience greater deterioration in quality of life; probably in agreement with the literature [52], these individuals experience greater long-term disabilities secondary to post-operative complications and thus long periods of hospitalization. Likewise, cancer survivors experience psychological problems such as depression, anxiety, and cognitive disabilities more frequently than the general population, which translates into greater difficulties in the reintegration of work and restoration of daily life rhythms enjoyed prior to the disease [29,53].

For the future, it would be desirable for cancer patients to be the protagonists of specific labor policies that take into account the possible impact of cancer pathology and related disabilities, including subsequent treatment, in order to guarantee their stable employment status, and encourage flexibility in terms of hours, tasks, and work intensity. Considering that in the absence of adequate support programs, employment rates decline substantially and the known impact of unemployment on health and mortality is even more burdensome, it will be important for future studies to assess the impact of unemployment itself on cancer.

Although all cancers and antineoplastic therapies have been found to be correlated with an increased risk of unemployment, it is clear that certain types of cancers (such as brain and colorectal cancers) and certain therapies (chemotherapy and radiation therapy) are more significantly correlated with disability in the short and long term, and thus with increased chances of job loss. Therefore, it is essential that more attention be paid to these types of patients, as well as female, obese, older and less educated individuals.

In addition, it would be preferable for patients to be supported with health and social welfare support programs, from diagnosis to follow-up, given the negative influence of cancer on every area of individuals’ lives. Therefore, it will be essential to consider the impact of individual treatment choices, thus seeking to increase patients’ involvement in decision-making.

## Figures and Tables

**Table 1:** Characteristics of selected studies.

Author and Year	Data Source	Country/Region	Sample Size	Years of Observation	Unemployment Measure Used	Population Characteristics	Primary Outcome
Yang SW. 2021 [26]	National Health Insurance Research Database. (NHIRD) Labor Insurance Database. (LID) Taiwan Cancer Registry. (TCR)	Taiwan	2451	5	Database	Employed: 1123 Unemployed: 1328	Return to work
Oh TK. 2022 [27]	National Health Insurance Service (NHIS) Statistics Korea	South Korea	4852	2	Database	Employed: 1415 Unemployed: 3437	Deterioration in quality of life, measures of unemployment
Senft C. 2020 [28]	Hospital	Germany	58	>3	Clinical data	Employed: 41 Unemployed: 17	Return to work
Alleaume C. 2018 [12]	French national survey VIe après le CANcer	France	969	5	Telephone interviews	Employed: 791 Unemployed: 178	Employment retention
Kiserud CE. 2016 [29]	Hospital	Norway	281	2	Questionnaire	Employed: 179 Unemployed: 102	Unemployment
Mannaerts GH. 2002 [30]	Hospital	Netherlands	76	5	Questionnaire	Employed: 21 Unemployed: 14 No job preoperatively: 39 Not answered: 2	Quality of life, return to work
Gregorowitsch ML. 2019 [31]	Netherlands Comprehesive Cancer Organization (IKNL)	Netherlands	Baseline: 939 30 nonths Follow-up: 319	2.5	Questionnaire	Baseline: Employed: 641 Unemployed: 298 30 Months Follow-up: Employed: 203 Unemployed: 116	Work ability, survival
Di Meglio A. 2020 [32]	Patients enrolled at 26 French cancer centers	France	1869	2	Questionnaire	Employed: 1471 Unemployed: 398	Return to work, unemployment

**Table 2:** RTI quality assessment of included studies. Y: Yes (affirmative answer). N: No (negative answer).

Author and Year	Q1	Q2	Q3	Q4	Q5	Q6	Q7	Q8	Q9	Q10	Q11	Q12	Q13	Q14	Q15	Q16	Overall Judgement
Yang SW. 2021 [26]	no	no	no	no	no	yes	No	no	no	No	yes	no	yes	no	no	No	High Quality
Oh TK. 2022 [27]	no	no	no	no	no	no	No	no	no	No	yes	no	yes	no	no	No	High Quality
Senft C. 2020 [28]	no	no	no	no	no	no	No	no	no	No	no	no	yes	yes	no	No	High Quality
Alleaume C. 2018 [12]	no	no	no	no	no	no	No	no	no	No	no	no	yes	no	no	No	High Quality
Kiserud CE. 2016 [29]	no	no	no	no	no	no	No	no	yes	No	no	yes	yes	no	no	No	High Quality
Mannaerts GH. 2002 [30]	no	no	no	no	no	no	No	no	no	No	no	no	yes	yes	no	No	High Quality
Gregorowitsch ML. 2019 [31]	no	no	no	no	no	no	No	no	no	No	no	no	yes	no	no	No	High Quality
Di Meglio A. 2020 [32]	no	no	no	no	no	no	No	no	no	No	no	no	yes	no	no	No	High Quality

**Table 3:** Summary table of cancers, therapies and induced disabilities.

Author and Year	Cancer Sites	Therapies	Induced Disabilities
Yang SW. 2021 [26]	Liver cancer (primary; I-II-III-IV stage)	Surgical treatment Radiotherapy Chemotherapy	Injuries related to chemotherapy: neutropenia, neuropathy, edema, nausea, cardiotoxicity, vomiting, fatigue
Oh TK. 2022 [27]	Brain tumor (Malignant)	Surgical treatment	Physical disability, 3.4% Brain disability, 17.8% Visual disability, 1.9% Hearing loss, 0.9% Speech disability, 0.4% Intellectual disorder, 0.6% Mental disorder, 0.2% Renal disability, 0.2%
Senft C. 2020 [28]	Brain tumor (grade II and III gliomas)	Surgical treatment, 20.7% Adjuvant therapy (chemotherapy or radiotherapy, 5.2%, both, 74.1%)	Fatigue, 60.8% Memory disturbances and difficulties concentrating, 43.1%, or finding words, 35.3%
Alleaume C. 2018 [12]	Breast cancer, 57.5% Lung aero-digestive tracts cancers, 7.1% Rectum/colon cancers, 6.2% Bladder/kidney/prostate cancers, 4.9% Thyroid cancer, 10.3% Hematological cancers (Non-Hodgkin Lymphoma), 3.4% Melanoma cancer, 6.8% Uterus and cervix cancers, 3.8%	Chemotherapy, 55.4% Radiotherapy, 65.9%	Chronic neuropathic pain, 30.1% Fatigue
Kiserud CE. 2016 [29]	Hematological cancers (Lymphoma: Hodgkin 25%, aggressive NHL 64%, indolent NHL 11%)	High-dose chemotherapy with autologous stem cell transplantation (HDT-ASCT)	Fatigue, 5.6% (physical fatigue 4.2%, mental fatigue 1.9%) Cognitive problems, 23% Anxiety, 4% Depression, 3.3% Negative affectivity, 39% Social inhibition, 38% Type D personality, 27%
Mannaerts GH. 2002 [30]	Rectal cancer (locally advanced primary, 49%, locally recurrent, 51%)	Surgical treatment Radiotherapy Multimodal treatment (preoperative radiotherapy, surgical treatment and intraoperative radiotherapy)	Fatigue, 44% Perineal pain, 42% Radiating leg pain, 21% Difficulty walking, 36% Urinary dysfunction, 42%
Gregorowitsch ML. 2019 [31]	Breast cancer	Systemic neoadjuvant therapy (chemotherapy or chemotherapy + immunotherapy) Radiotherapy Surgical treatment Adjuvant chemotherapy Adjuvant endocrine	Physical impairment Mental impairment
Di Meglio A. 2020 [32]	Breast cancer (stage I-II-III)	Surgical treatment (partial, 69.9%, mastectomy, 30.1%) Axillary surgery (sentinel lymph node. 53.9%, axillary dissection, 46.1%) Adjuvant radiotherapy, 91.5% Neo adjuvant chemotherapy, 65.6% Adjuvant endocrine therapy, 82.4% Adjuvant anti-HER2 therapy, 15.1%	Anxiety, 38.2% Depression, 6.9% Weight changes (≥5%, 44.3%)

## Appendix A

**Table A1:** PICO query items.

PICO	
Population	Employed adults aged between 18 and 65 years, in a post-cancer follow-up of at least 2 years.
Exposure	Any regimen option among:
	- no treatment/palliative care - surgery/radiotherapy - chemotherapy/immunotherapy
Comparison	Different therapy regimens
Outcome	Change in employment status: - same employment - job reallocation - unemployed - anticipated retirement and/or sick leave

**Table A2:** Search strategies.

Database	Search String(s)
Medline	(cancer[MeSH Terms] OR neoplasm[Title/Abstract] OR carcinoma[Title/Abstract] OR tumor[Title/Abstract] OR oncology[Title/Abstract]) AND (radiotherapy[Title/Abstract] OR chemotherapy[Title/Abstract] OR immunotherapy[Title/Abstract] OR surgery[Title/Abstract]) AND (employment[Title/Abstract] OR unemployment[Title/Abstract] OR retirement[Title/Abstract] OR sick leave[Title/Abstract] OR sickness absence[Title/Abstract] OR absenteeism[Title/Abstract] OR presenteeism[Title/Abstract] OR work[Title/Abstract] OR occupation[Title/Abstract] OR work ability[Title/Abstract] OR work disability[Title/Abstract] OR disability management[Title/Abstract] OR rehabilitation[Title/Abstract] OR vocational[Title/Abstract]) AND (survivor[Title/Abstract] OR survival[Title/Abstract] OR long-term[Title/Abstract] OR mortality[Title/Abstract] OR dead[Title/Abstract])
Scopus	(cancer OR carcinoma) AND (treatment OR therapy) AND (employment OR unemployment)
